# Dataset of multiple methodology characterization of an illite-kaolinite clay mineral for the purpose of using it as ceramic membrane supports

**DOI:** 10.1016/j.dib.2020.105300

**Published:** 2020-02-18

**Authors:** Abdelaziz Elgamouz, Najib Tijani, Ihsan Shehadi, Kamrul Hasan, Mohamad Al-Farooq Kawam

**Affiliations:** a Department of Chemistry, College of Sciences, Research Institute of Science and Engineering, University of Sharjah, P.O. Box 27272, Sharjah, United Arab Emirates; b Group: Membranes, Matériaux et Procédés de Séparation, Faculté des Sciences, Université Moulay Ismaîl, Meknès, Morocco

**Keywords:** Membrane supports, Methylene blue test, Clay minerals, Ultrafiltration, Pores structures, Desalination

## Abstract

This article describes the data generated from multiple approach methodology physico-chemical characterization of a clay mineral from the West-Central region of Morocco, Safi province (https://doi.org/10.1016/j.heliyon.2019.e02281) [1]. Data were generated from classical chemical analytical techniques namely; organic matter content, linear firing and shrinkage analysis, weight loss on ignition, porosity and methylene blue stain tests according to the French Association of Normalization (AFNOR) and American Society for Testing and Materials (ASTM). In addition to data generated using instrumental analytical techniques namely; Infrared spectroscopy (FTIR), thermal gravimetric analysis (TGA) and deferential thermal analysis (DTA), X-ray diffraction (XRD), scanning electron microscopy (SEM) and elemental energy disperse spectroscopy (EDX).

**Table:** Specifications Table

Subject area	*6.1: Chemistry: Analytical Chemistry*
More specific subject area	*Surface analysis, phase analysis*
Type of data	Table, figure and image (blue stain test)
How data was acquired	• ARL-8660 X-Ray Fluorescence Spectrometer SETARAM TGA instrument • Bruker Platinum ATR tensor II FTIR spectrometer • NABER 2804 furnace • X-ray diffraction (XRD) using a D-Max Rigaku X-ray diffractometer with a copper anode and a graphite monochromator to select CuK_α1_ radiation (λ = 1.540 Å). • T*escan* VEGA XM variable pressure SEM equipped with Oxford Instruments X-Max 50 EDX detector, controlled with AZtecEnergy analysis software with a resolution of 125 eV to determine the abundance of elements.
Data format	Raw, filtered, analyzed
Parameters for data collection	• Two clay samples (SA) and (CH) were crushed to coarse material, then to fine powder followed by sieving using standardized AFNOR sieves in the range 250–500 μm. • Clay flat discs (membrane supports) were prepared from 250 to 500 μm granulometry to obtain pellets with a diameter of 25.0 mm and a thickness of 2.0 mm. • Supports were calcined to final temperatures ranging from 250 °C to 1100 °C. These were used to measure shrinkage/dilatation, chemical resistance and water porosity. • Clay powder was used to extract the clay fraction, HCl was used to destroy the carbonates and help extract the clay fractions. While ammonia (NH3) was used to promote deflocculating. • ASTM and AFNOR methylene blue tests were used to characterize the clay fractions and specific surface area of the clay samples.
Description of data collection	The tow clay samples (SA) and (CH) were characterized by various methods: XRD, SEM-EDX, FTIR, TGA/TDA and later semi-quantitatively found the concentration of the pure clay fractions (illite and kaolinite) using the blue stain test according to AFNOR and ASTM.
Data source location	Safi/West region/Atlantic coast/Morocco 10 Km from Safi center. Latitude: 32° 16′ 60.00″ N, Longitude: −9° 13′ 60.00″ W.
Data accessibility	The data represented is with this article.
Related research article	Abdelaziz Elgamouz, Najib Tijani, Ihsan Shehadi, Kamrul Hasan, Mohamad Al-Farooq Kawam, Characterization of the firing behaviour of an illite-kaolinite clay mineral and its potential use as membrane support, Heliyon, 5 (2019) [1].

**Table:** 

**Value of the data** • These data are relevant to ceramic membrane support fabrication, especially fabrication of membrane supports from clay minerals. • The data would allow other researchers to identify the key parameters that need to be controlled when investigating new clay material. • These data gave a detailed and complete set of experiments that could be used in the characterization of widely available depot of clays and provide an insight on how clay fractions could be characterized and how these could affect the quality of the final products. • The data reveal new ways in which largely available clay minerals could be used to develop sustainable and cheap clay supports.

## 1 Data

**Fig. 1:**
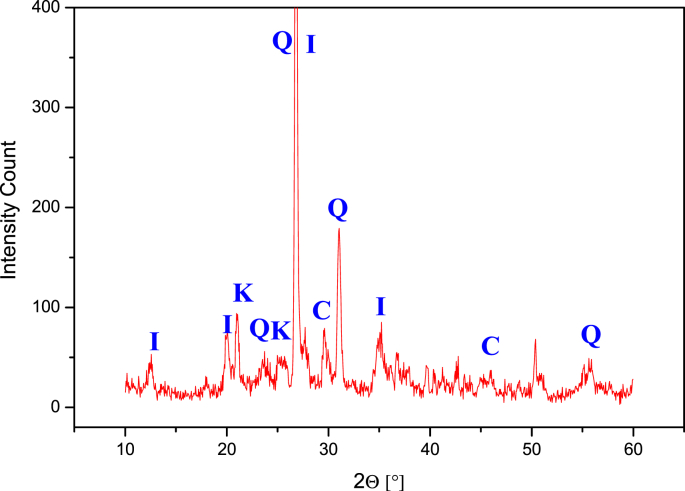
X-ray diffraction for SA crude clay sample. Q: Quartz, C: Calcite, I: Illite and K: Kaolinite.

**Fig. 2:**
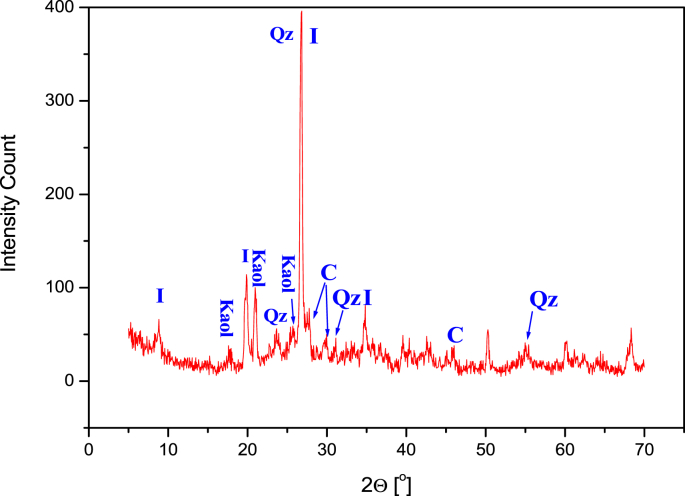
X-ray diffraction for CH crude clay sample. Q: Quartz, C: Calcite, I: Illite and K: Kaolinite.

**Fig. 3:**
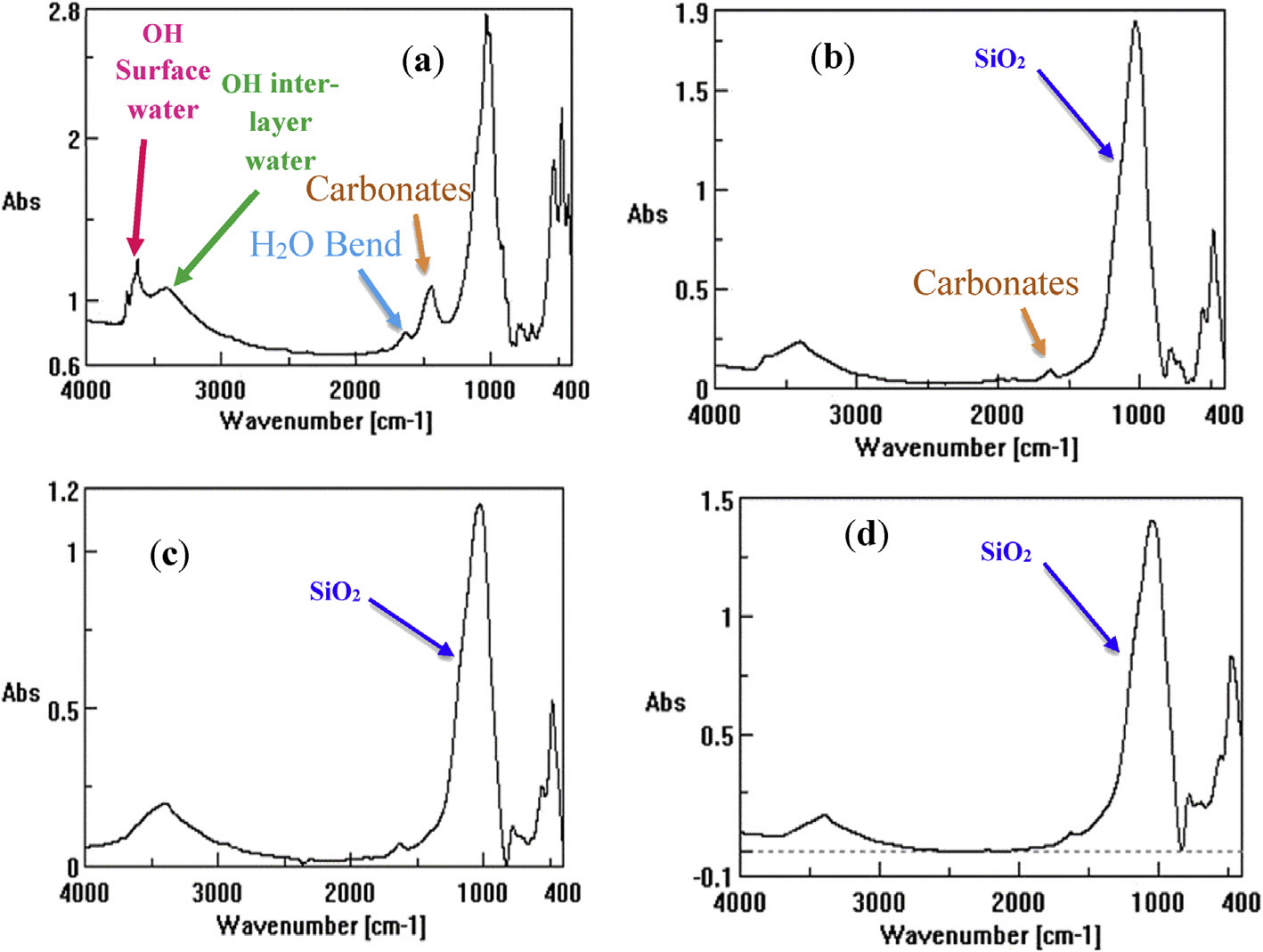
FTIR spectrum of the SA natural clay sample at different temperatures showing the main vibrations, (a) crude clay, (b) calcined to 250 °C, (c) calcined to 500 °C and (d) calcined to 850 °C.

**Fig. 4:**
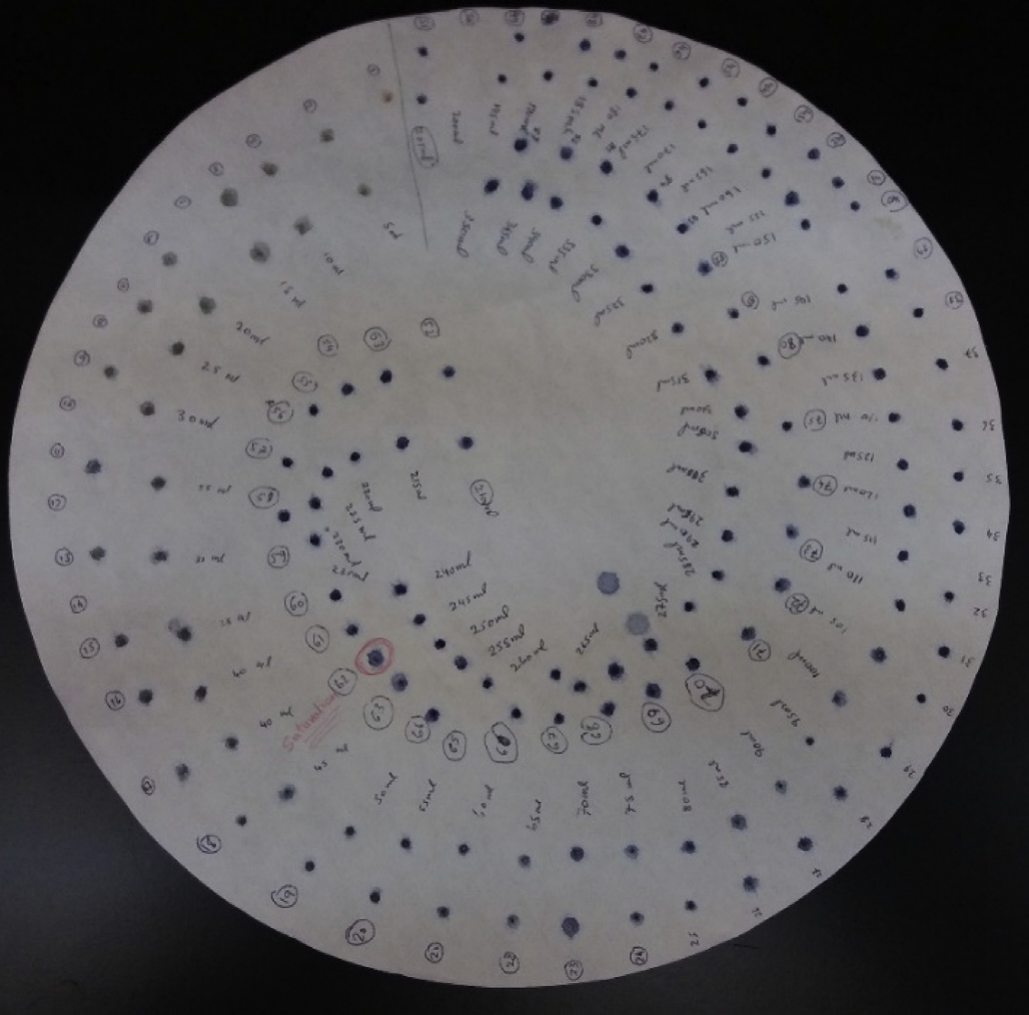
Blue stains (spots) spotted on a Whatman 1441–055 Quantitative Filter Paper Circles, 20 Micron, Grade 41, 55mm Diameter.

Successive weight loss on ignition for both clay powder samples (SA) and (CH) at final temperatures of 250 °C, 500 °C, 700 °C, 800 °C, 900 °C and 1000 °C are presented in Table 1. While, data for the weight loss of compacted clay discs at final Temperatures of 500 °C, 700 °C, 800 °C, 850 °C, 900 °C, 950 °C, 1000 °C, 1050 °C and 1100 °C are presented in Table 2. The Shrinkage analysis data for the compacted flat discs (SA) and (CH) at final temperature of 500 °C, 700 °C, 800 °C, 850 °C, 900 °C, 950 °C, 1000 °C, 1050 °C and 1100 °C are presented in Table 3. The data for water porosity of the fabricated clay supports calcined at final temperatures of 500 °C, 700 °C, 800 °C, 900 °C and 1000 °C are presented in Table 4. The data of chemical resistance is presented as weight loss fraction (Δm/m_o_) of SA and CH compacted flat discs calcined to 850 °C and treated with HCl at pH = 5 and NaOH at pH = 10.0. The elemental chemical composition of crude and calcined clays SA and CH at 850 °C and 950 °C are given in Table 6, Table 7, Table 8, Table 9, Table 10, Table 11. Fig. 1, Fig. 2 represent the X-ray diffraction of the crude samples SA and CH respectively. The interreticular measured distances, the Miller indices and the 2θ position of the diffractometric reflects X-ray diffraction for all phases are given in Table 12. Fig. 3 represent SA clay FTIR spectra at final temperatures of 250 °C, 500 °C and 850 °C, CH sample was not shown because of similarities with SA sample while FTIR peak attributions are given in Table 13. Fig. 4 represents the blue stains test spotted on a Whatman Filter Paper, 55mm Diameter. Thermal phenomenon observed during the linear calcination of the SA clay powder are presented in Table 14.

**Table 1:** Successive loss on ignition for SA and CH clays powders calcined from 25 °C to final temperatures of 250 °C, 500 °C, 700 °C, 800 °C, 900 °C and 1000 °C at rate of 5 °C/min.

	SA	CH	Loss difference (SA)	Loss difference (CH)
Calcined to 250°C	4.12 ± 0.19	3.74 ± 0.27	4.12 ± 0.19	3.74 ± 0.27
Calcined to 500°C	5.23 ± 0.09	5.05 ± 0.13	1.11 ± 0.21	1.31 ± 0.30
Calcined to 700°C	11.26 ± 0.09	11.17 ± 0.13	6.03 ± 0.13	6.12 ± 0.18
Calcined to 800°C	11.52 ± 0.06	11.33 ± 0.07	0.26 ± 0.11	0.16 ± 0.14
Calcined to 900°C	12.38 ± 0.05	12.28 ± 0.07	0.87 ± 0.08	0.95 ± 0.10
Calcined to 1000°C	12.69 ± 0.07	12.60 ± 0.07	0.31 ± 0.09	0.31 ± 0.10

**Table 2:** Loss on ignition for SA and CH clays compacted flat discs calcined from 25 °C to final temperatures of 500 °C, 700 °C, 800 °C, 850 °C, 900 °C, 950 °C, 1000 °C, 1050 °C and 1100 °C at rate of 5 °C/min.

	SA (g)	CH (g)	Δm/m_o_ (CH)	Δm/m_o_ (CH)
Before Calcination	3.0900 ± 0.0001	3.0000 ± 0 .0001	–	–
Calcined to 500°C	2.8800 ± 0.0001	2.7400 ± 0.0001	8.83 ± 0.10	6.77 ± 0.04
Calcined to 700°C	2.6600 ± 0.0001	2.5300 ± 0.0001	15.65 ± 0.09	13.74 ± 0.04
Calcined to 800°C	2.6700 ± 0.0001	2.5400 ± 0.0001	15.59 ± 0.09	13.72 ± 0.04
Calcined to 850°C	2.6400 ± 0.0001	2.5100 ± 0.0001	16.38 ± 0.08	14.49 ± 0.03
Calcined to 900°C	2.6400 ± 0.0001	2.5100 ± 0.0001	16.41 ± 0.08	14.46 ± 0.03
Calcined to 950°C	2.6400 ± 0.0001	2.5100 ± 0.0001	16.53 ± 0.08	14.64 ± 0.03
Calcined to 1000°C	2.6300 ± 0.0001	2.5000 ± 0.0001	16.61 ± 0.08	14.75 ± 0.03
Calcined to 1050°C	2.6300 ± 0.0001	2.5000 ± 0.0001	16.65 ± 0.08	14.79 ± 0.03
Calcined to 1100°C	2.6300 ± 0.0001	2.5000 ± 0.0001	16.81 ± 0.08	14.85 ± 0.03

**Table 3:** Shrinkage on ignition for SA and CH clays compacted flat discs calcined from 25 °C to final temperatures of 500 °C, 700 °C, 800 °C, 850 °C, 900 °C, 950 °C, 1000 °C, 1050 °C and 1100 °C at rate of 5 °C/min.

	SA (mm)	CH (mm)	ΔL/L_o_ (SA)	ΔL/L_o_ (CH)
Before Calcination	30.42 ± 0.02	30.42 ± 0.02	–	–
Calcined to 500°C	30.41 ± 0.02	30.39 ± 0.02	0.03 ± 0.01	0.10 ± 0.01
Calcined to 700°C	30.40 ± 0.02	30.37 ± 0.02	0.07 ± 0.02	0.16 ± 0.01
Calcined to 800°C	30.38 ± 0.02	30.35 ± 0.02	0.13 ± 0.02	0.23 ± 0.01
Calcined to 850°C	30.16 ± 0.02	30.14 ± 0.02	0.85 ± 0.05	0.92 ± 0.08
Calcined to 900°C	29.92 ± 0.02	30.00 ± 0.02	1.64 ± 0.06	1.38 ± 0.08
Calcined to 950°C	29.76 ± 0.02	29.86 ± 0.02	2.17 ± 0.06	1.84 ± 0.08
Calcined to 1000°C	29.72 ± 0.02	29.80 ± 0.02	2.30 ± 0.08	2.04 ± 0.07
Calcined to 1050°C	29.70 ± 0.02	29.78 ± 0.02	2.37 ± 0.02	2.10 ± 0.06
Calcined to 1100°C	29.42 ± 0.02	29.49 ± 0.02	3.29 ± 0.01	3.06 ± 0.08

**Table 4:** Water porosity for SA and CH clays compacted flat discs calcined from at final temperatures of 500 °C, 700 °C, 800 °C, 900 °C, and 1000 °C at rate of 5 °C/min.

	SA (g) before immersing in water	CH (g) before immersing in water	SA (g) after drying	CH (g) after drying	SA-Porosity (%)	CH-Porosity (%)
Before Calcination	3.0033 ± 0.0001	3.0894 ± 0.0001	–	–	–	–
Calcined to 500°C	2.7382 ± 0.0001	2.8804 ± 0.0001	3.2985 ± 0.0001	2.7820 ± 0.0001	20.12 ± 0.48	18.23 ± 0.47
Calcined to 700°C	2.5333 ± 0.0001	2.6649 ± 0.0001	3.0620 ± 0.0001	2.5717 ± 0.0001	23.46 ± 0.66	21.61 ± 0.60
Calcined to 800°C	2.5351 ± 0.0001	2.6655 ± 0.0001	3.0156 ± 0.0001	2.5714 ± 0.0001	18.39 ± 0.80	16.72 ± 0.79
Calcined to 900°C	2.5105 ± 0.0001	2.6428 ± 0.0001	2.9462 ± 0.0001	2.5433 ± 0.0001	17.58 ± 0.32	16.06 ± 0.31
Calcined to 1000°C	2.5045 ± 0.0001	2.6336 ± 0.0001	2.8581 ± 0.0001	2.5346 ± 0.0001	16.00 ± 0.65	14.62 ± 0.62

**Table 5:** Weight loss after treatment with HCl and NaOH for SA and CH clays compacted flat discs calcined at 850 °C.

	SA (g) before	CH (before)	SA (g) after	CH (g) after	Δm/m_o_ (SA)	Δm/m_o_ (CH)
pH = 5.0	3.0033 ± 0.0001	3.0894 ± 0.0001	2.3767 ± 0.0001	2.4400 ± 0.0001	20.86 ± 0.03	21.02 ± 0.04
pH = 10.0	3.0109 ± 0.0001	3.0100 ± 0.0001	2.300 ± 0.01	2.1021 ± 0.0001	23.61 ± 0.01	30.16 ± 0.06

**Table 6:** Percentages of the oxides composing the SA crude clay powder.

Elem	Wt %	Mol %	K-Ratio	Z	A	F
Na_2_O	1.42	1.6	0.0028	0.967	0.2776	1.0046
MgO	4.48	7.76	0.0107	0.992	0.3954	1.0084
Al_2_O_3_	25.24	17.3	0.0662	0.9634	0.5101	1.009
SiO_2_	51.88	60.33	0.1209	0.992	0.5021	1.0013
SO_3_	1.37	1.19	0.0029	0.9851	0.5436	1.0031
Cl_2_O	0.49	0.39	0.0024	0.9364	0.6477	1.0048
K_2_O	4.54	3.36	0.029	0.9404	0.8145	1.0057
CaO	3.7	4.61	0.0216	0.964	0.8458	1.0029
TiO_2_	0.99	0.87	0.0048	0.8839	0.9158	1.0053
Fe_2_O_3_	5.9	2.58	0.0361	0.8841	0.99	1.0
Total	100	100

**Table 7:** Percentages of the oxides composing the CH crude clay powder.

Elem	Wt %	Mol %	K-Ratio	Z	A	F
Na_2_O	0.96	0.85	0.0017	0.9514	0.2552	1.0024
MgO	6.34	2.97	0.0048	0.976	0.3699	1.0043
Al_2_O_3_	12.92	6.94	0.0326	0.9479	0.5	1.0049
SiO_2_	25.44	23.18	0.0653	0.9762	0.5619	1.0008
SO_3_	0.46	0.31	0.0012	0.9695	0.6787	1.0023
K_2_O	2.66	1.55	0.0187	0.9229	0.9102	1.0052
CaO	2.42	2.36	0.0153	0.9466	0.9324	1.0026
TiO_2_	0.5	0.33	0.0024	0.8685	0.9752	1.0053
Fe_2_O_3_	4.15	1.42	0.0255	0.8684	1.014	1
Total	100	100

**Table 8:** Percentages of the oxides composing the SA clay powder calcined to 850 °C.

Elem	Wt %	Mol %	K-Ratio	Z	A	F
Na_2_O	0.83	2.04	0.0034	0.9703	0.2589	1.0042
MgO	4	12.34	0.0157	0.9953	0.3674	1.0071
Al_2_O_3_	22.37	15.46	0.0534	0.9666	0.4632	1.0079
SiO_2_	48.61	57.02	0.1092	0.9953	0.4823	1.001
SO_3_	1.37	1.19	0.0029	0.9851	0.5436	1.0031
Cl_2_O	0.49	0.39	0.0024	0.9364	0.6477	1.0048
K_2_O	4.32	3.23	0.0279	0.9441	0.82	1.0066
CaO	3.29	4.14	0.0195	0.9676	0.8516	1.0049
TiO_2_	0.5	0.44	0.0025	0.8871	0.9212	1.0112
Fe_2_O_3_	4.12	5.32	0.0742	0.8874	0.993	1.0
Total	100	100

**Table 9:** Percentages of the oxides composing the CH clay powder calcined to 850 °C.

Elem	Wt %	Mol %	K-Ratio	Z	A	F
Na_2_O	0.85	0.73	0.0016	0.9514	0.2672	1.003
MgO	7.09	9.41	0.0162	0.9759	0.3871	1.0046
Al_2_O_3_	10.29	5.4	0.0252	0.9478	0.4857	1.0059
SiO_2_	31.11	27.71	0.0802	0.9761	0.5647	1.0009
SO_3_	0.47	0.31	0.0012	0.9694	0.6566	1.0023
K_2_O	2.76	1.57	0.019	0.9229	0.8966	1.0051
CaO	3.01	2.87	0.0188	0.9466	0.9212	1.0015
TiO_2_	0.47	0.31	0.0024	0.8685	0.966	1.0025
Fe_2_O_3_	2.03	0.68	0.0125	0.8683	1.0105	1
Total	100	100

**Table 10:** Percentages of the oxides composing the SA clay powder calcined to 950 °C.

Elem	Wt %	Mol %	K-Ratio	Z	A	F
Na_2_O	0.82	0.73	0.0015	0.9521	0.2607	1.0027
MgO	2.7	3.71	0.006	0.9766	0.3783	1.0049
Al_2_O_3_	18.6	7.87	0.037	0.9485	0.506	1.0055
SiO_2_	49.96	26.74	0.0739	0.9768	0.5575	1.0007
SO_3_	1.37	1.19	0.0029	0.9851	0.5436	1.0031
Cl_2_O	0.49	0.39	0.0024	0.9364	0.6477	1.0048
K_2_O	2.63	1.55	0.0182	0.9236	0.9004	1.0048
CaO	2.44	2.41	0.0153	0.9473	0.925	1.0022
TiO_2_	0.5	0.35	0.0025	0.8691	0.9706	1.0042
Fe_2_O_3_	3.37	1.17	0.0207	0.869	1.0123	1.0
Total	100	100

**Table 11:** Percentages of the oxides composing the CH clay powder calcined to 950 °C.

Elem	Wt %	Mol %	K-Ratio	Z	A	F
Na_2_O	0.91	0.82	0.0017	0.9562	0.2646	1.0033
MgO	9	12.54	0.0205	0.9809	0.3824	1.0049
Al_2_O_3_	11.6	6.39	0.0276	0.9526	0.4684	1.0064
SiO_2_	34.19	31.96	0.085	0.981	0.5413	1.0012
SO_3_	0.55	0.38	0.0013	0.9742	0.6294	1.0032
K_2_O	3.61	2.15	0.0246	0.9284	0.879	1.0077
CaO	5.09	5.1	0.0313	0.952	0.902	1.0019
TiO_2_	0.47	0.31	0.0024	0.8685	0.966	1.0025
Fe_2_O_3_	4.43	1.56	0.0271	0.8733	1.0032	1
Total	100	100

**Table 12:** Interreticular measured distances, the Miller indices and the 2θ position of the diffractometric reflects X-ray diffraction for all phases.

d_hkl_	(*hkl*)	2 Theta	(*hkl*)	phase
4.27	(100)	20.9	(100)	Quartz
3.35	(101)	26.7	(101)	Quartz
2.45	(110)	36.5	(110)	Quartz
2.12	(200)	42.5	(200)	Quartz
1.81	(112)	50.1	(112)	Quartz
1.373	(212)	67.6	(212)	Quartz triplet
1.374	(203)	67.7	(203)	
1.380	(301)	68.07	(301)	
7.17	(001)	12.3	(001)	Kaolinite
4.47	(020)	19.8	(020)	Kaolinite
3.57	(002)	24.8	(002)	Kaolinite
2.38	(003)	37.9	(003)	Kaolinite
10.0	(002)	8.7	(002)	Illite
5.02	(004)	17.6	(004)	Illite
3.34	(006)	26.6	(006)	Illite
3.84	(012)	23.1	(012)	Calcite
3.04	(104)	29.4	(104)	Calcite
2.83	(113)	39.5	(113)	Calcite

**Table 13:** Infrared peak attributions for SA crude clay mineral and calcined to final temperature of 850 °C (CH spectra was similar).

IR Frequencies of crude clay	IR Frequencies of calcined clay to 850 °C	Attributions
3708	Disappeared because of calcination	ν Si–OH external (SiO_2_)
3632	3660	ν Al–OH external (Al_2_O_3_)
3406	3412	ν OH (H_2_O) interlayer water
1625	1640	δ OH(OH2) [2]
1437	Disappeared because of calcination	ν CO3
1031	1034	ν Si–O (SiO_2_)
909	Disappeared because of calcination	δ(Al.Si.Mg.Ca)OH
870	Disappeared because of calcination	δ(CO3)
786	Disappeared because of calcination	δ(CO3)

ν: stretching vibration, δ: bending vibration.

**Table 14:** Thermal analysis (TGA/TDA) attribution of different phenomena of the SA and CH clay samples.

TGA weight losses' intervals (^o^C)	TDA Peaks	Phenomenon	Attribution
30–123	90	endothermic	departure of the water adsorbed on surface of the clay
366–402	390	endothermic	Interlayer water departure
465–587	530	endothermic	Kaolinite and illite decompositions
674–760	740	endothermic	Calcite decomposition
908–935	925	exothermic	Mullite crystallization

## 2 Experimental design, materials, and methods

### 2.1 Equations used in classical method analysis

#### 2.1.1 Weight loss on ignition

(1) Mass loss % =(m110−mTm110)×100

In Eq. (1), m_110_ is the specimen's weight at 110 °C, m_T_ is the specimen's weight fired at final temperature of 250, 500, 700, 800, 850, 900, 950, 1000, 1050 and 1100 °C.

#### 2.1.2 Shrinkage analysis

(2) ΔL/L0=(LT−L0L0)×100

In Eq. (2), L_0_ is the diameter of the flat disc before firing and L_T_ is the diameter flat disc after calcination to final temperature T.

#### 2.1.3 Water absorption and porosity

(3) P % =(mf−m0m0)×100

In Eq. (3), m_0_ is the initial weight and m_f_ is the finale weight of the flat disc after firing.

#### 2.1.4 Chemical resistance

(4) $$R=\frac{m_{o}-m_{pH,10}}{m_{o}}$$

In Eq. (4) m_o_ is the mass of the flat disc before pH attack and m_pH_, is its mass after removing the disc from the acidic (HCl, pH = 5.0) or basic (NaOH, pH = 10.0) solutions for 24 hours.

#### 2.1.5 Blue value (AFNOR)

The blue value of the clay following AFNOR procedure was calculated using Eq. (5).

(5) VB=(V×0.01×100)M

In Eq. (5), V defines the methylene blue volume flowed in mL, 0.01 is the concentration in g/mL of the methylene blue solution, and M is the mass in grams of the dry sample.

#### 2.1.6 Blue index (ASTM)

The blue index according to ASTM (MIB, in equivalence/100 g) was calculated following Eq. (6) [3].

(6) MIB=(E×V×0.1)W

In Eq. (6), E stands for the MB number of equivalents per mL of water, V represents the volume of the MB solution in mL (unit of titration was = 1.0 mL), and W represents the weight of the dry clay sample in g.

### 2.2 Loss on ignition

The loss on ignition was carried out on clay powders, for this 3.0 g of crude clays (SA or CH) where introduced into porcelain crucibles and successively heated to final temperatures of 250 °C, 500 °C, 700 °C, 800 °C, 900 °C and 1000 °C at heating rate of 5 °C/min following the heating program derived from TGA/TDA analysis represented below.

(7) $$25^{o}C\overset{5^{o}C/min}{\to }250^{o}C\left(hold2h\right)\overset{5^{o}C/min}{\to }500^{o}C\left(hold2h\right)\overset{5^{o}C/min}{\to }700^{o}C\left(hold2h\right)\overset{5^{o}C/min}{\to }T_{f}$$

While the loss on ignition on specimen was carried out on flat disc made by compacting 3.0 g of the SA or CH clay powders with the granulometry of 250–500 μm made by ASTM standardized sieves and under a uniaxial pressure $4.6\times 10^{7}Pa$. The obtained pellets with diameter of 25.0 mm and thickness of 2.0 mm were calcined to various final temperatures between 250 °C and 1100 °C following the same heating program described above.

### 2.3 Shrinkage analysis

For shrinkage analysis, the weight before calcination and after calcination to a final temperature of previously compacted flat discs were recorded and calculations were made according to Eq. (2).

### 2.4 Water absorption and porosity

Flat discs compacted and calcined to temperature equal or above 500 °C were immersed in degassed boiled distilled water for a period of 24 h, the discs were removed and dried in the open air for a period of 10 min then weighed and water porosity of the specimen were determined using Eq. (3).

### 2.5 Chemical resistance

The resistance of SA and CH compacted flat discs to drastic acidic or basic conditions were tested by measuring the weight loss of the two specimen after immersing them in HCl solution of pH = 5.0 and NaOH solution of pH = 10.0. The weight losses under acidic and based condition were determined using Eq. (4).

### 2.6 Oxides composition of the clay minerals

The elemental chemical analysis of the natural clay was performed by a *Tescan* VEGA-XM SEM spectrometer equipped with Oxford Instruments X-Max 50 EDX detector. The EDX measurements were acquired from the characteristic peaks of elements present in the clay (Na, K, Mg, Ca, Al, Si, S, Ti and Fe). Elements concentrations were determined after treatment of signals and the main outcome of the analysis is the (K-ratio). The concentration of the i-th element in the sample was calculated using Eq. (5) [4,5].

(8) $$\frac{C_{i}}{C_{\left(i\right)}}=ZAF\times \frac{I_{i}}{I_{\left(i\right)}}=ZAF\times K-ratio$$

The oxides concentration was calculated by using the “Elements by difference” in advanced pan EDX software, oxygen concentration was calculated as difference between 100% and the sum of all other elements. Then, percentages of oxides were calculated by combining oxygen with all other elements that can form oxides [6]. Based on previous studies on clay oxides used were: Na_2_O, MgO, Al_2_O3, SiO_2_, SO_3_, Cl_2_O, K_2_O, CaO, TiO_2_ and Fe_2_O_3_ [7,8]. The compositions of the two clay samples SA and CH in oxides at different temperatures are given in Table 5, Table 6, Table 7, Table 8, Table 9, Table 10.

### 2.7 X-ray diffraction

The clay powder was analyzed using XRD, two types of analysis were carried out on the clay samples. First, non-oriented analysis of fine enough powders was carried out in order to ensure the homogeneity of the sample. Second, 2.0 μm deposited clay fraction on a glass slide, which was extracted using the protocol for clay fraction extraction described later and left to dry for 24 hours at room temperature. In both analysis, non-oriented and oriented fraction, data was recorded using Cu_Kα_ radiation (λ = 1.540 Å) on a PW1710 Philips Analytical diffractometer controlled by XPERT Quantify software (EA Almelo, The Netherlands) operating at 40 kV and 30 mA, with a copper anode and a graphite monochromator with a normal divergence (1.0°) and receiving slits of 0.1 mm dimensions. 1300 points were recorded using continuous scans in a 2θ range of 5.0° to 80°.

### 2.8 Clay fraction isolation

To separate elements with fraction less than 2 μm oriented preparation were made in 6 different steps; **i**- **Grinding**, it must produce a powder which is not too fine to preserve the clay minerals with a diameter of less than 2 μm [9]. **ii- Discharge**, it is done with distilled water, then the sample is subjected to magnetic stirring. The coarse material deposited at the bottom of the flask is removed by decanting. **iii- Decarbonation**, this involves the removal of carbonates (CaCO_3_). This operation is necessary for several reasons [10], i) the carbonates include the clay minerals, and hence interfere with their deflocculating; ii) they dilute the clay fraction; obstruct the orientation of the preparations by their non-lamellar form. Hydrochloric acid diluted to 10% is added dropwise to the clay suspension with magnetic stirring in order to avoid local overconcentration, while allowing a little time between each attack. pH is controlled with pH meter. When the solution becomes red, this indicate that the carbonates have been destroyed, at this stage it is necessary to stop the HCl addition and the agitation, then the suspension was allowed to settle. **iv- Washing,** its purpose is to free the sample from the excess of the hydrochloric acid, and to allow deflocculating of the clay fraction. If the supernatant becomes clear, it is enough to wash the sample without using the centrifuge, meaning, pour the supernatant, add distilled water, shake, leave it to decant, and so on until a neutral pH is obtained, pH was controlled by litmus paper. Otherwise, the suspensions were centrifuged at 2500 rpm for 5 min. Subsequently, the supernatant is removed, and the precipitate is re-suspended in distilled water. The precipitate is recovered, tested with pH paper, if it is not yet neutral, the centrifugation cycles must be re-instated until the pH is neutral. **v- Suspended Sample**, the recovered precipitate is placed in a Beaker of 250 ml, to which distilled water is added, manually shaken, and left to decant for 1h 40mn. If, after 10 minutes, the supernatant is clear. This indicates that deflocculating is poorly performed. For this purpose, one to two drops of ammonia (NH_4_OH) are added which reduces the pH to around 7.0 (the color of the pH paper becomes blue), this is an indication that deflocculating is promoted. **vi- Extraction of particles smaller than 2μm**, the supernatant must be cloudy so that the suspension is perfect. The contents of the upper 2 cm of the supernatant are recovered and placed in a Beaker of 100 mL. Distilled water was added and centrifuged at 3500 rpm for 40 minutes. The precipitate obtained is recovered by means of a spatula, deposited in glass slides, left to dry for 24 hours at room temperature and finally passed to analysis by X-ray diffraction.

### 2.9 Infrared spectroscopy

The FTIR analysis was carried out in the spectral range (400–4000) cm^−1^ by a Bruker Platinum ATR tensor II spectrometer with a resolution of 4 cm^−1^. A fine clay powder, raw or calcined to the required final temperature was kept in the oven at 70 °C, then taken in a desiccator to analysis in order to ensure absorbed waters are not present in the clay samples. The sample was squeezed between the swivel pressure tower and the diamond crystal of the ATR unit, then IR beam was stricken into the sample. Data was recorded in the transmittance mode. Only infrared of SA samples were recorded CH show identical spectra with SA, therefore was not shown.

### 2.10 Methylene blue (MB) stain test

Methylene blue stain test according to AFNOR was carried out using the following procedure [11]; 60.0 g of the clay sample was suspended in 500 mL of distilled water and stirred vigorously until homogenization. 5.0 mL of 10.0g/L of methylene blue solution were added to the homogenized solution using a burette. After each addition, spots (stains) were spotted on a Whatman 1441–055 Quantitative Filter Paper Circles, 20 μm, Grade 41, 55mm Diameter using a capillary. The sampling and spotting continued until the wet surface surrounding the deep blue spot is turned into light blue color. This represents the saturation of the clay by methylene blue.

## References

[1] Elgamouz A., Tijani N., Shehadi I., Hasan K., Al-Farooq Kawam M. (2019). Characterization of the firing behaviour of an illite-kaolinite clay mineral and its potential use as membrane support. Heliyon.

[2] Saikia B.J., Parthasarathy G. (2010). Fourier transform infrared spectroscopic characterization of kaolinite from Assam and Meghalaya, Northeastern India. J. Mod. Phys..

[3] ASTM (2009). Standard Test Method for Methylene Blue Index of Clay.

[4] Wassilkowska A., Czaplicka-Kotas A., Bielski A., Zielina M. (2014). An analysis of the elemental composition of micro-samples using EDS technique. Czasopismo Techniczne.

[5] Goldstein J.I., Newbury D.E., Michael J.R., Ritchie N.W., Scott J.H.J., Joy D.C. (2017). Scanning Electron Microscopy and X-Ray Microanalysis.

[6] Konopka J. (2013). Options for Quantitative Analysis of Light Elements by SEM/EDS.

[7] Elgamouz A., Tijani N. (2018). Dataset in the production of composite clay-zeolite membranes made from naturally occurring clay minerals. Data in brief.

[8] Elgamouz A., Tijani N. (2018). From a naturally occurring material (clay mineral) to the production of porous ceramic membranes. Microporous Mesoporous Mater..

[9] Lahcen Daoudi, DeVleeschouwer, François, Richard Bindler, El Ouahabi Meriam, Fagel Nathalie (2014). Potentials of using Moroccan clays in the ceramic industry: the case of the deposits of Jbel Kharrou and Benhmed (Western Moroccan Meseta). JMMCE.

[10] Ostrom M.E. (1961). Separation of clay minerals from carbonate rocks by using acid. J. Sediment. Res..

[11] French Association of Normalization, (ANFOR) (1993). Measurement of the Quantity and Activity of the Clay Fraction (French Standard NF P 94-068).

